# Association between enthesitis/dactylitis resolution and patient-reported outcomes in guselkumab-treated patients with psoriatic arthritis

**DOI:** 10.1007/s10067-024-06921-8

**Published:** 2024-03-12

**Authors:** Proton Rahman, Iain B. McInnes, Atul Deodhar, Georg Schett, Phillip J. Mease, May Shawi, Daniel J. Cua, Jonathan P. Sherlock, Alexa P. Kollmeier, Xie L. Xu, Shihong Sheng, Christopher T. Ritchlin, Dennis McGonagle

**Affiliations:** 1 Memorial University of Newfoundland, St. Johns, NF Canada; 2 University of Glasgow, Glasgow, UK; 3 Oregon Health & Science University, Portland, OR USA; 4 FAU Erlangen-Nürnberg and Universitätsklinikum, Erlangen, Germany; 5 Rheumatology Research, Providence Swedish Medical Center, Seattle, WA USA; 6 University of Washington School of Medicine, Seattle, WA USA; 7 Janssen Research & Development, LLC, Titusville, NJ USA; 8 Janssen Research & Development, LLC, Spring House, PA USA; 9 University of Oxford, Oxford, UK; 10 Janssen Research & Development, LLC, San Diego, CA USA; 11 University of Rochester Medical Center, Rochester, NY USA; 12 Leeds Biomedical Research Centre, University of Leeds, 2nd Floor, Chapel Allerton Hospital, Chapeltown Road, Leeds, LS7 4SA UK

**Keywords:** Biologic, Dactylitis, Enthesitis, Guselkumab, Psoriatic arthritis

## Abstract

**Objectives:**

To evaluate the association between enthesitis resolution (ER) and dactylitis resolution (DR) and meaningful improvements in patient-reported outcomes (PROs) among biologic-naïve patients with PsA receiving guselkumab in the DISCOVER-2 study.

**Methods:**

Enthesitis and dactylitis, characteristic lesions of PsA, were evaluated by independent assessors using the Leeds Enthesitis Index (range, 0–6) and Dactylitis Severity Score (range, 0–60). Proportions of patients with ER or DR (score = 0) among those with score > 0 at baseline were determined at weeks 24, 52, and 100. PROs included: fatigue (Functional Assessment of Chronic Illness Therapy-Fatigue [FACIT-Fatigue]), pain (0–100 visual analog scale), physical function (Health Assessment Questionnaire-Disability Index [HAQ-DI]), and health-related quality of life (36-item Short-Form Health Survey physical/mental component summary [SF-36 PCS/MCS]). Meaningful responses were defined as: improvements of ≥ 4 for FACIT-Fatigue, ≥ 0.35 for HAQ-DI, and ≥ 5 for SF-36 PCS/MCS and absolute scores of ≤ 15 for minimal pain and ≤ 0.5 for normalized HAQ-DI. Associations between ER/DR status and PRO response status were tested using a Chi-square test.

**Results:**

Guselkumab-treated patients with ER were more likely than those without ER to achieve minimal pain (*p* < 0.001), normalized HAQ-DI (*p* < 0.001), and PCS response (*p* < 0.05) at weeks 24, 52, and 100. Patients with DR were more likely than those without DR to achieve FACIT-Fatigue response at week 24 and week 52 (both *p* ≤ 0.01) and minimal pain at week 24 and normalized HAQ-DI at week 52 (both *p* ≤ 0.03).

**Conclusion:**

In biologic-naïve patients with active PsA treated with guselkumab, achieving ER or DR was associated with durable improvements in selected PROs, including those of high importance to patients.

**Trial registration:**

ClinicalTrials.gov (https://clinicaltrials.gov) NCT03158285; Registered: May 16, 2017.

**Table:** 

**Key Points**
• *At week 100, 65% and 76% of guselkumab-treated patients achieved enthesitis and dactylitis resolution (ER/DR).* • *Achieving ER was associated with achieving DR and vice versa through the end of study.* • *Achieving ER or DR was associated with durable and meaningful improvements in selected patient-reported outcomes.*

**Supplementary Information:**

The online version contains supplementary material available at 10.1007/s10067-024-06921-8.

## Introduction

Psoriatic arthritis (PsA) is an inflammatory musculoskeletal and cutaneous disease with heterogenous clinical presentation and variable course. Musculoskeletal manifestations include enthesitis and dactylitis along with peripheral arthritis and axial disease often accompanied by the dermatologic manifestations of psoriasis and nail lesions [1–3]. These hallmark features of PsA should be considered separately in a disease domain-based approach to treatment [4, 5]. Enthesitis is defined as inflammation of tendon, ligament, or joint capsule insertion sites to bone, while dactylitis is defined as diffuse swelling of a digit resulting in sausage-shaped fingers or toes and constituted by inflammation of joints, extra-articular connective tissues, and bone in that digit [6, 7]. These conditions occur variably in up to half of all patients with PsA (35–50% [enthesitis] and 16–50% [dactylitis]) [2, 3, 7]. Both enthesitis and dactylitis are associated with more severe disease and are often difficult to treat, thereby producing significant disease burden for PsA patients and challenges to clinicians treating patients with these manifestations [8, 9].

Patient-reported outcomes (PROs) provide critical information beyond core disease measures for determining the most effective management of PsA, as perceptions of disease severity and importance of specific symptoms frequently differ between patients and physicians [10]. Impaired health-related quality of life (HRQoL) and physical function are well documented in patients with PsA, including those with enthesitis and dactylitis [11–13]. Furthermore, patients identify relief from pain and fatigue as high treatment priorities [14, 15] and the levels of both pain and fatigue are higher in patients with concomitant enthesitis and/or dactylitis than in those without these manifestations [8, 9, 16].

Guselkumab, a fully human monoclonal antibody with specificity to the IL-23 p19 subunit, is approved for the treatment of adults with active PsA. In patients with active disease enrolled in DISCOVER-1 (both biologic-naïve and -experienced patients) and DISCOVER-2 (biologic-naïve patients), significant improvements in the signs and symptoms of PsA were noted at week 24 following treatment with guselkumab 100 mg administered either every 4 weeks (Q4W) or at weeks 0 and 4 and then every 8 weeks (Q8W) compared with placebo [17, 18]. Durable efficacy was observed across multiple PsA domains, including enthesitis and dactylitis, through week 52 and week 100 [19–21]. Independent of one another, resolution of enthesitis and dactylitis as well as benefit to selected PROs were reported through 2 years in DISCOVER-2 [21–23].

Enthesitis and dactylitis frequently co-occur and may share common pathogenetic features [6]. In these post hoc analyses, we assessed associations between resolution of enthesitis and dactylitis among guselkumab-treated patients in the DISCOVER-2 study. Also, the correlations between resolution of enthesitis and/or dactylitis and meaningful improvements in PROs especially relevant in PsA (i.e., fatigue, pain, function, and HRQoL) were evaluated in guselkumab-treated patients through the end (2 years) of the DISCOVER-2 study.

## Methods

### Patients and study design

The DISCOVER-2 study (clinicaltrials.gov: NCT03158285) was a phase 3, multicenter, randomized, double-blind, placebo-controlled study conducted to assess the efficacy and safety of guselkumab in biologic-naïve patients with active PsA. Patients were randomized (1:1:1) to receive subcutaneous injections of guselkumab 100 mg at weeks 0, 4, then every Q4W (Q4W group); guselkumab 100 mg at weeks 0, 4, then Q8W (Q8W group); or placebo with crossover to guselkumab 100 mg Q4W at week 24 (i.e., placebo crossover group). The final study drug administration and efficacy assessments occurred at week 100.

Patient eligibility criteria and study design details have been published previously [18]. Briefly, patients had active PsA (≥ 5 swollen joints, ≥ 5 tender joints, and C-reactive protein ≥ 0.6 mg/dL) despite treatment with standard therapies (i.e., non-biologic disease-modifying antirheumatic drugs [DMARDs], apremilast, or nonsteroidal anti-inflammatory drugs [NSAIDs]). Patients were allowed to continue stable doses of selected standard treatments (e.g., NSAIDs, methotrexate, and corticosteroids).

This study was conducted in accordance with the principles of the Declaration of Helsinki and Good Clinical Practices; the study protocols were approved by an Institutional Review Board or Ethics Committee at each site, and all patients provided written informed consent.

### Assessments

Independent assessors determined the presence of enthesitis and/or dactylitis using the Leeds Enthesitis Index (LEI) and Dactylitis Severity Score (DSS), respectively. The LEI assesses the absence or presence (0 or 1) of tender entheses in the left and right lateral epicondyle, medial femoral condyle, and Achilles tendon insertion (total score range: 0–6) [24]. For the DSS, each of 20 digits of the hands and feet are evaluated for swelling and erythema on a scale of 0–3 (0 = absent, 1 = mild, 2 = moderate, 3 = severe) for a total score of 0–60 (i.e., 0–30 for hands and 0–30 for feet) [25]. Enthesitis resolution (ER) and dactylitis resolution (DR) were defined as a baseline LEI or DSS score > 0 and post-baseline visit score = 0.

Data on patients’ self-evaluation of fatigue were collected using the Functional Assessment of Chronic Illness Therapy-Fatigue (FACIT-Fatigue; range, 0–52; higher scores indicate less fatigue); response is defined as a change (increase) in FACIT-Fatigue score of ≥ 4 points [26]. Pain level was self-assessed using a 0–100 unit visual analog scale (VAS), with a value ≤ 15 considered to be minimal pain as described in the PsA Minimal Disease Activity (MDA) response criteria [27]. To assess physical function, patients completed the Health Assessment Questionnaire-Disability Index (HAQ-DI) to quantitate their difficulty with daily activities based on 20 function-specific questions (score: 0 [indicating no difficulty] to 3 [indicating inability to perform a task in that area]; lower scores are indicative of better functioning) [28]. Achieving an improvement (decrease) of ≥ 0.35 points is considered a clinically meaningful HAQ-DI response in PsA while attaining an overall score ≤ 0.5 indicates normalized physical function [27, 29]. Health-related quality of life was determined by physical component summary (PCS) and mental component summary (MCS) scores on the 36-item Short-Form Health Survey (SF-36; range, 0–100); for both, higher scores indicate better health status, and response was defined as a change (increase) ≥ 5 points [30, 31].

### Data analyses

Demographic and disease characteristics were summarized for patients with and without enthesitis, with and without dactylitis, and with and without both conditions at baseline. Efficacy outcomes through week 100 are reported for each randomized treatment group or for the combined guselkumab group, which includes both the Q8W and Q4W groups.

Least squares (LS) mean of changes in LEI and DSS at weeks 24, 52, and 100 among patients with enthesitis and dactylitis, respectively, at baseline were determined by analysis of covariance; explanatory factors were treatment group, baseline score, and randomization factors (i.e., baseline usage of non-biologic DMARD [yes, no] and the most recently available CRP value prior to randomization [< 2.0, ≥ 2.0 mg/dL]). Through week 24, patients who met treatment failure (TF) criteria were considered as having no change from baseline (i.e., change set to 0) and had remaining missing data imputed by multiple imputations (MI) under the assumption of missing at random (MAR). After week 24, for patients who discontinued the study agent for any reason, the change from baseline, if missing, was set to 0 and then, for all patients, the remaining missing data were imputed by MI under the assumption of MAR. Proportions of patients achieving ER (defined as LEI = 0) and DR (defined as DSS = 0) were determined among patients with enthesitis and dactylitis, respectively, at baseline. In the analyses of proportions of patients achieving ER/DR, patients who met TF criteria or had missing data for any reason were considered as not achieving ER or DR through week 24. No TF rule was applied after week 24; patients with missing data for any reason were classified as not achieving ER or DR through week 100 [18, 20, 21].

Associations between ER and DR and associations between ER or DR with a PRO binary endpoint were analyzed based on observed data (i.e., data for which no TF rule was applied and no missing data were imputed). The PRO binary response endpoints were improvements of ≥ 4-point increase for FACIT-Fatigue response; ≥ 0.35-point decrease (among patients with baseline score ≥ 0.35) for HAQ-DI response; and ≥ 5-point increase for SF-36 PCS and MCS response and absolute scores of ≤ 15 (on a 100-unit VAS) for minimal pain and ≤ 0.5 (among patients with baseline score > 0.5) for normalized HAQ-DI. Correlations between ER or DR status and a PRO binary response endpoint were tested using a Chi-square test or, in the case of rare events, using a Fisher’s exact test; all *p*-values were considered nominal.

## Results

### Patient characteristics

Demographic and disease characteristics for the 739 randomized and treated patients (Q4W *n* = 245, Q8W *n* = 248, placebo *n* = 246) in DISCOVER-2 were generally well balanced across treatment groups [18]. For the full study population, overall measures of disease activity were consistent with active PsA. Across treatment groups, mean scores for FACIT-Fatigue (range: 29–31), pain (62–63), HAQ-DI (1.2–1.3) and SF-36 PCS (32–33) and MCS (47–48) indicated the patient-reported health status was impaired in this population of biologic-naïve patients with active PsA [18, 32].

Approximately 69% of patients enrolled in DISCOVER-2 had enthesitis (*n* = 506; mean LEI: 2.8) and 45% had dactylitis (*n* = 331; mean DSS: 8.3) at baseline (Table 1). Dactylitis was more common in patients with enthesitis vs. without (51% vs. 32%) (Table 2). Likewise, enthesitis was more common in patients with dactylitis vs. without (78% vs. 61%). Among patients with enthesitis, severe LEI scores (≥ 3) were seen in 52% of patients with dactylitis and 44% without dactylitis.

**Table 1:** Baseline characteristics for patients with and without enthesitis or dactylitis enrolled in the DISCOVER-2 study

	Enthesitis		Dactylitis		Enthesitis & Dactylitis	
	With	Without	With	Without	With	Without^a^
Patients, n (%)	506 (68.6)	232 (31.4)	331 (44.9)	407 (55.1)	258 (35.0)	480 (65.0)
Demography						
Male	257 (50.8)	131 (56.5)	193 (58.3)	195 (47.9)	151 (58.5)	237 (49.4)
Age (years)	45.3 ± 11.6	46.4 ± 11.9	44.4 ± 11.4	46.7 ± 11.8	44.5 ± 11.2	46.3 ± 11.9
Weight (kg)	84.3 ± 20.3	84.2 ± 17.7	83.7 ± 20.0	84.8 ± 19.1	83.9 ± 20.5	84.5 ± 19.0
BMI (kg/m^2^)	29.1 ± 6.5	28.6 ± 5.5	28.4 ± 6.0	29.4 ± 6.3	28.5 ± 6.1	29.2 ± 6.2
Normal (< 25)	145 (28.7)	62 (26.7)	104 (31.4)	103 (25.3)	83 (32.2)	124 (25.8)
Overweight (≥ 25- < 30)	161 (31.8)	88 (37.9)	107 (32.3)	142 (34.9)	79 (30.6)	170 (35.4)
Obese (≥ 30)	200 (39.5)	82 (35.3)	120 (36.3)	162 (39.8)	96 (37.2)	186 (38.8)
PsA disease characteristics						
PsA duration (years)	5.4 ± 5.8	5.6 ± 5.6	5.3 ± 5.7	5.6 ± 5.7	5.4 ± 5.8	5.5 ± 5.7
SJC (0–66)	12.9 ± 7.8	10.9 ± 5.5	14.4 ± 8.2	10.5 ± 5.7	15.1 ± 8.7	10.8 ± 5.7
TJC (0–68)	23.9 ± 13.7	15.4 ± 8.2	25.1 ± 14.2	18.2 ± 10.8	27.2 ± 14.6	18.1 ± 10.6
CRP, mg/dL	2.2 ± 2.7	1.6 ± 1.8	2.2 ± 2.7	1.83 ± 2.2	2.3 ± 2.8	1.8 ± 2.2
Median (IQR)	1.3 (0.6–2.7)	1.0 (0.6–2.2)	1.4 (0.7–2.7)	1.0 (0.5–2.3)	1.5 (0.7–2.7)	1.0 (0.6–2.3)
LEI score (1–6)	2.8 ± 1.6	N/A	3.0 ± 1.7	2.7 ± 1.5	3.0 ± 1.7	2.7 ± 1.5
DSS (1–60)	9.1 ± 10.0	5.8 ± 6.9	8.3 ± 9.5	N/A	9.1 ± 10.0	5.8 ± 6.9
PsA-modified vdH-S (0–528)	25.4 ± 39.8	23.1 ± 38.3	30.3 ± 45.6	20.1 ± 32.7	30.3 ± 44.1	21.7 ± 36.2
Median (IQR)	10.5 (4.0–27.0)	10.3 (3.5–25.3)	13.5 (4.5–31.0)	9.0 (3.0–22.5)	14.0 (4.5–31.5)	9.5 (3.5–23.3)
PASDAS (0–10)	6.9 ± 1.0	6.0 ± 0.9	7.3 ± 1.0	6.1 ± 0.8	7.4 ± 0.9	6.2 ± 0.8
DAPSA^b^	51.8 ± 21.1	40.5 ± 14.7	54.8 ± 22.2	42.9 ± 16.2	57.8 ± 22.8	43.1 ± 16.2
cDAPSA^c^	49.6 ± 20.8	38.9 ± 14.4	52.6 ± 21.8	41.1 ± 16.1	55.4 ± 22.5	41.3 ± 16.0
PsO disease characteristics						
IGA (0–4)						
< 2	83 (16.4)	50 (21.6)	51 (15.4)	82 (20.1)	37 (14.3)	96 (20.0)
≥ 2	423 (83.6)	182 (78.4)	280 (84.6)	325 (79.9)	221 (85.7)	384 (80.0)
PASI (0–72)	10.7 ± 11.7	8.2 ± 9.4	10.7 ± 11.9	9.3 ± 10.4	11.3 ± 12.0	9.2 ± 10.5
< 12	359 (70.9)	182 (78.4)	238 (71.9)	303 (74.4)	179 (69.4)	362 (75.4)
≥ 12	147 (29.1)	50 (21.6)	93 (28.1)	104 (25.6)	79 (30.6)	118 (24.6)
Percent BSA, n	504	232	331	405	258	478
< 3%	85 (16.9)	51 (22.0)	53 (16.0)	83 (20.5)	37 (14.3)	99 (20.7)
≥ 3%	419 (83.1)	181 (78.0)	278 (84.0)	322 (79.5)	221 (85.7)	379 (79.3)
Patient-reported outcomes						
SF-36 PCS (0–100)	32.1 ± 7.2	34.2 ± 7.4	32.5 ± 7.1	33.0 ± 7.5	32.2 ± 6.7	33.1 ± 7.6
SF-36 MCS (0–100)	47.2 ± 11.2	48.7 ± 11.3	47.3 ± 11.1	47.9 ± 11.5	46.5 ± 10.8	48.3 ± 11.5
FACIT-Fatigue (0–52)	28.9 ± 9.5	31.6 ± 9.9	29.0 ± 9.6	30.3 ± 9.8	28.2 ± 9.1	30.6 ± 9.9
HAQ-DI (0–3)	1.3 ± 0.6	1.1 ± 0.6	1.4 ± 0.6	1.2 ± 0.6	1.4 ± 0.5	1.2 ± 0.6
Pain (0–100 VAS)^d^	62.8 ± 18.4	61.5 ± 20.5	64.5 ± 19.0	60.8 ± 19.0	65.1 ± 18.3	61.0 ± 19.3
Concomitant medication, n	506	232	331	407	258	480
csDMARDs	357 (70.6)	154 (66.4)	246 (74.3)	265 (65.1)	192 (74.4)	319 (66.5)
MTX	312 (61.7)	130 (56.0)	213 (64.4)	229 (56.3)	168 (65.1)	274 (57.1)
Other^e^	45 (8.9)	24 (10.3)	33 (9.9)	36 (8.8)	24 (9.3)	45 (9.3)
NSAIDs	343 (67.8)	160 (69.0)	215 (65.0)	288 (70.8)	159 (61.6)	344 (71.7)

Data are mean ± SD or number of patients (%), unless otherwise indicated. BMI, body mass index; BSA, body surface area affected by PsO; cDAPSA, clinical DAPSA (without CRP); CRP, C-reactive protein; csDMARDs, conventional synthetic disease-modifying antirheumatic drugs; DAPSA, Disease Activity in Psoriatic Arthritis; DSS, Dactylitis Severity Score; FACIT-Fatigue, Functional Assessment of Chronic Illness Therapy–Fatigue; HAQ-DI, Health Assessment Questionnaire-Disability Index; IGA, Investigator’s Global Assessment; IQR, interquartile range; LEI, Leeds Enthesitis Index; MCS, mental component summary; MTX, methotrexate; NSAIDs, nonsteroidal anti-inflammatory drugs; PASDAS, Psoriatic Arthritis Disease Activity Score; PASI, Psoriasis Area and Severity Index; PCS, physical component summary; PsA, psoriatic arthritis; PsO, psoriasis; SD, standard deviation; SF-36, 36-Item Short Form Health Survey; SJC, swollen joint count; TJC, tender joint count; VAS, visual analog scale; vdh-S, van der Heijde-Sharp

^a^Patients in the ‘Without Enthesitis and Dactylitis’ group did not have both conditions but could have had one or the other

^b^DAPSA scores indicate disease activity level as follows: 0–4 (remission), 5–14 (low), 15–28 (moderate) and > 28 (high)

^c^cDAPSA scores indicate disease activity level as follows: 0–4 (remission), 5–13 (low), 14–27 (moderate) and > 27 (high)

^d^Pain was measured using a 100-unitVAS scale as part of minimal disease activity (MDA), defined as achievement of 5 of the following 7 criteria: TJC ≤ 1, SJC ≤ 1, PASI score ≤ 1, patient pain VAS ≤ 15, patient global disease activity VAS ≤ 20, HAQ-DI ≤ 0.5, and tender entheseal points ≤ 1

^e^Other concomitant medications include hydroxychloroquine, sulfasalazine, and leflunomide

**Table 2:** Proportion of patients with and without dactylitis and/or enthesitis at baseline by treatment group

	Guselkumab Q4W	Guselkumab Q8W	Placebo	Total
Patients, N	245	248	246	739
Patients with dactylitis, N	121	111	99	331
Patients with enthesitis (+ / +)^a^	95 (78.5)	82 (73.9)	81 (81.8)	258 (77.9)
LEI score = 1	23 (24.7)	22 (26.8)	15 (19.0)	60 (23.6)
LEI score = 2	19 (20.4)	23 (28.0)	19 (24.1)	61 (24.0)
LEI score ≥ 3	51 (54.8)	37 (45.1)	45 (57.0)	133 (52.4)
Patients without dactylitis, N	124	137	146	407
Patients with enthesitis (-/ +)^a^	75 (60.5)	76 (55.5)	97 (66.4)	248 (60.9)
LEI score = 1	18 (24.7)	19 (25.3)	26 (27.1)	63 (25.8)
LEI score = 2	19 (26.0)	22 (29.3)	32 (33.3)	73 (29.9)
LEI score ≥ 3	36 (49.3)	34 (45.3)	38 (39.6)	108 (44.3)
Patients with enthesitis, N^b^	170	158	178	506
Patients with dactylitis (+ / +)	95 (55.9)	82 (51.9)	81 (45.5)	258 (51.0)
Patients without enthesitis, N	75	90	67	232
Patients with dactylitis (-/ +)	26 (34.7)	29 (32.2)	18 (26.9)	73 (31.5)

LEI, Leeds Enthesitis Index; Q4W, every 4 weeks; Q8W, every 8 weeks

Data presented as n (%) unless otherwise noted. ^a^8 patients with missing LEI score; ^b^8 patients with missing LEI score; + / + represents patients with both enthesitis and dactylitis; -/ + represents patients without dactylitis with enthesitis or patients without enthesitis with dactylitis

Baseline patient characteristics were generally consistent between those with enthesitis and those with dactylitis and between those with and without each condition, although patients with either condition had, on average, greater levels of disease activity as assessed by composite measures. While demographic characteristics were generally similar across patients with both enthesitis and dactylitis at baseline (*n* = 258; 35%) and those with neither or only one of the two manifestations, several numerical differences are noted (Table 1). More males had both conditions (59%) vs. neither condition or just one of the conditions (49%), driven by the higher proportion of males with dactylitis. When assessed by individual and composite measures, patients with both enthesitis and dactylitis generally had more active joint and skin disease at baseline than those without both conditions (Table 1). Specifically, mean PASDAS scores were 7.4 in patients with enthesitis and dactylitis, and 6.2 in those without both conditions; corresponding mean DAPSA scores were 58 and 43. Additionally, patients with both conditions had more pre-existing structural damage, as evidenced by the numerically higher van der Heijde-Sharp score (median 14.0 vs. 9.5), and were more likely to have more severe skin disease (Psoriasis Area and Severity Index ≥ 12). Among mean PRO scores at baseline, FACIT-Fatigue and SF-36 were comparable between patients with and without both conditions, whereas pain and HAQ-DI scores were numerically higher in patients with vs. without both conditions.

Concomitant use of conventional synthetic DMARDS was somewhat more common in patients with both enthesitis and dactylitis (74%) than in those without either condition (67%). The proportion of patients using concomitant NSAIDs was lower for those with (62%) vs. without (72%) both conditions, mainly driven by differential use in patients with (65%) vs. without (71%) dactylitis in the context of comparable use in patients with (68%) vs. without (69%) enthesitis.

#### Patient disposition

Patient disposition for the overall DISCOVER-2 population has been reported through week 100 [20, 21]. In patients with enthesitis or dactylitis at baseline, retention rates were robust (89% [448/506] and 88% [292/331], respectively) and consistent with those reported for all patients (88% [652/741]) through the end of the study.

### Guselkumab effects on enthesitis, dactylitis, and PROs

Least squares mean changes from baseline in LEI scores were greater in the guselkumab Q4W/Q8W groups than in the placebo group at week 24 (-1.5/-1.6 vs. -1.0) and long-term effect was observed in guselkumab-treated patients through week 100; the same was true for LS mean changes from baseline in DSS (Fig. S1) [20, 21]. Furthermore, in the guselkumab groups, response rates for achieving either ER or DR were higher than those in the placebo group at week 24, increased through week 52, and were maintained through week 100 (Fig. S2). Meaningful improvements in FACIT-Fatigue, pain, HAQ-DI, and/or SF-36 PCS/MCS scores have also been reported for guselkumab-treated patients over time [18, 20, 21, 33].

### Associations between resolution of enthesitis or dactylitis with guselkumab

**Fig. 1:**
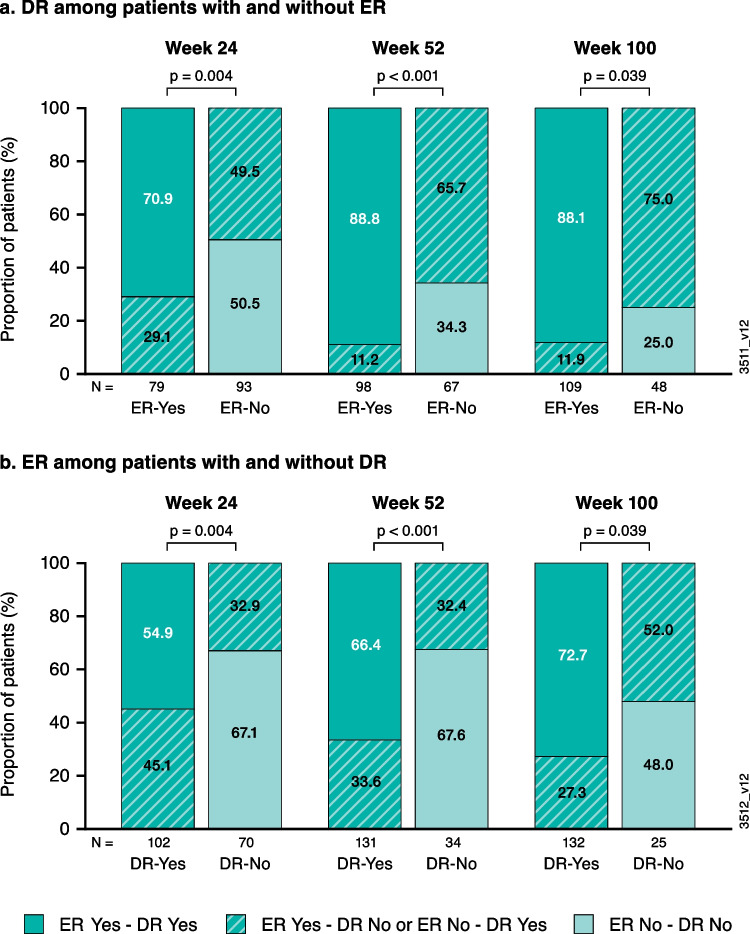
Proportion of patients achieving ER or DR among those with and without resolution of the other based on observed data at weeks 24, 52, and 100: **a**) DR among patients who did vs. did not achieve ER; and **b**) ER among patients who did vs. did not achieve DR. DR, dactylitis resolution; ER, enthesitis resolution

Patients who achieved ER were more likely to also achieve DR and vice versa at weeks 24, 52, and 100 (all *p* < 0.05; Fig. 1**)**. Among patients who achieved ER, 55% (week 24), 66% (week 52), and 73% (week 100) also achieved DR at the corresponding timepoints; among those achieving DR at weeks 24, 52, and 100, ER was also achieved by 71%, 89%, and 88%, respectively.

### Associations between resolution of enthesitis or dactylitis and PROs

#### Fatigue and pain

**Fig. 2:**
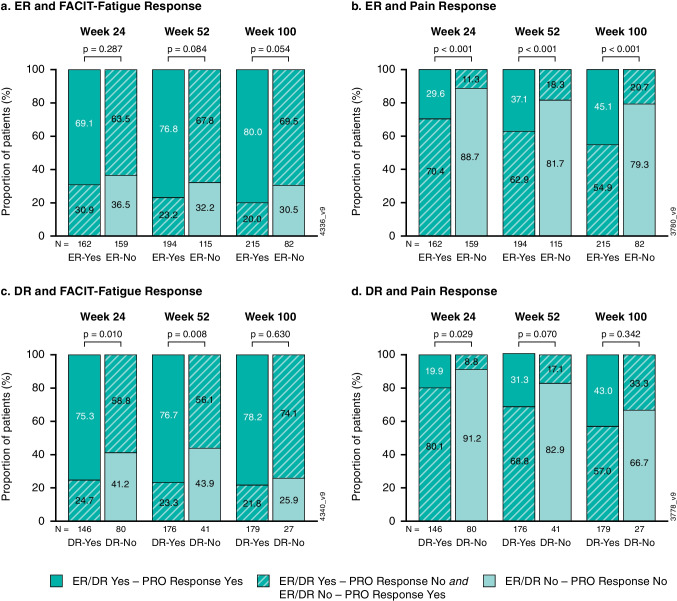
Proportion of patients achieving fatigue response and minimal pain among those with vs. without ER or DR based on observed data at weeks 24, 52, and 100: Proportion of patients achieving fatigue and minimal pain responses among those with and without ER or DR based on observed data at weeks 24, 52, and 100: **a**) FACIT-Fatigue response and **b**) minimal pain response in patients with vs. without ER; **c**) FACIT-Fatigue response and **d**) minimal pain response in patients with vs. without DR. DR, dactylitis resolution; ER, enthesitis resolution; FACIT-Fatigue, Functional Assessment of Chronic Illness Therapy-Fatigue; FACIT-Fatigue response, ≥ 4 point improvement; pain response, ≤ 15 on 100-unit visual analog scale; PRO, patient-reported outcome

Among guselkumab-treated patients with enthesitis at baseline, > 60% of patients achieved FACIT-Fatigue response at week 24 regardless of whether or not they achieved ER. Response rates for achieving FACIT-Fatigue response were numerically higher among patients achieving ER than those not achieving ER at week 52 (77% vs 68%) and week 100 (80% vs. 70%) (Fig. 2a). Patients achieving ER were more likely to achieve minimal pain response than those not achieving ER at week 24 (30% vs. 11%), week 52 (37% vs. 18%), and week 100 (45% vs. 21%; all *p* < 0.001; Fig. 2b).

Among guselkumab-treated patients with dactylitis at baseline, those achieving DR were more likely than those not achieving DR to achieve FACIT-Fatigue response at week 24 (75% vs. 59%) and week 52 (77% vs. 56%) (Fig. 2c) and minimal pain response at week 24 (20% vs. 9%; all *p* < 0.05; Fig. 2d). Proportions of guselkumab-treated patients achieving these endpoints at weeks 52 and 100 were numerically higher among those with DR.

#### Physical function

**Fig. 3:**
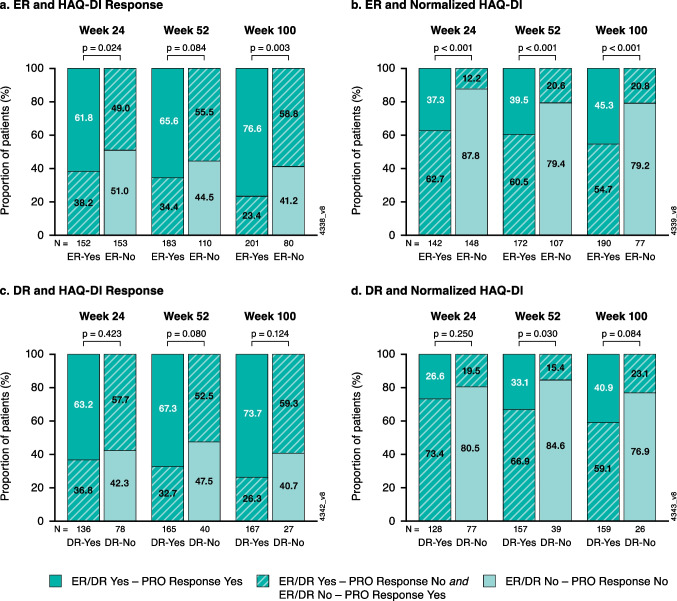
Proportion of patients achieving physical function response among those with vs. without ER or DR based on observed data at weeks 24, 52, and 100: **a**) HAQ-DI response and **b**) normalized HAQ-DI in patients with vs. without ER; **c**) HAQ-DI response and **d**) normalized HAQ-DI in patients with vs. without DR. DR, dactylitis resolution; ER, enthesitis resolution; HAQ-DI, Health-Assessment Questionnaire-Disability Index; HAQ-DI response, ≥ 0.35 point improvement in HAQ-DI score; normalized HAQ-DI, score of ≤ 0.5; PRO, patient-reported outcome

Relative to patients not achieving ER, those achieving ER were more likely to achieve HAQ-DI response at weeks 24 (62% vs. 49%) and 100 (77% vs. 59%); both *p* < 0.05; at week 52, the HAQ-DI response rate was numerically higher among those achieving ER at week 52 (66% vs. 56%; *p* = 0.084) (Fig. 3a). Likewise, patients achieving ER were more likely (*p* < 0.001) to achieve normalized HAQ-DI than those without ER at weeks 24 (37% vs. 12%), 52 (40% vs. 21%), and 100 (45% vs. 21%) (Fig. 3b).

The proportions of patients achieving HAQ-DI response were numerically higher among those with (63–74%) vs. without (53–59%) DR over time (Fig. 3c**)**. Similarly, numerically higher proportions of patients achieved DR and normalized HAQ-DI at each timepoint, with a significant association at week 52 (33% vs. 15%, *p* < 0.030) (Fig. 3d).

#### Health-related quality of life

**Fig. 4:**
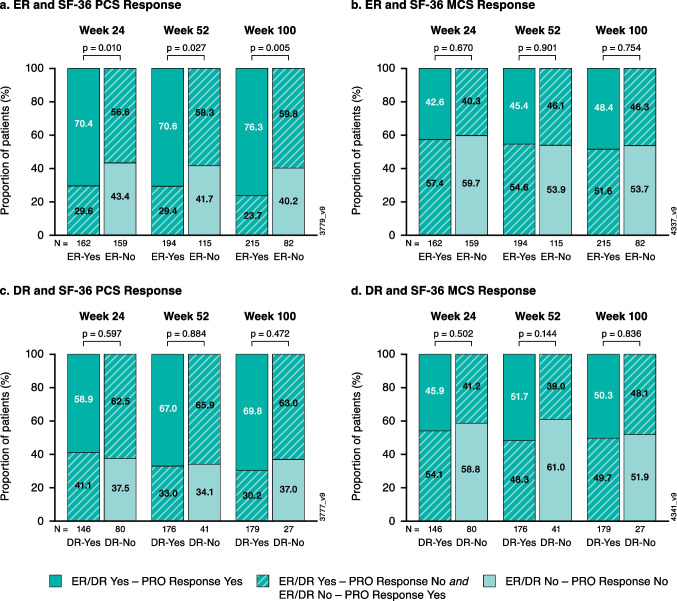
Proportion of patients achieving HRQoL response among those with vs. without ER or DR based on observed data at weeks 24, 52, and 100: **a**) SF-36 PCS response and **b**) SF-36 MCS response in patients with vs. without ER; **c**) SF-36 PCS response and **d**) SF-36 MCS response in patients with vs. without DR. DR, dactylitis resolution; ER, enthesitis resolution; HRQoL, health-related quality of life; MCS, mental component summary; PCS, physical component summary; PRO, patient-reported outcome; SF-36, 36-item Short-Form Health Survey; SF-36 PCS response, ≥ 5 point improvement in SF-36 PCS; SF-36 MCS response, ≥ 5 point improvement in SF-36 MCS

Patients who achieved ER were more likely to achieve SF-36 PCS response compared with those who did not achieve ER at week 24 (70% vs. 57%); the association was maintained through week 52 (71% vs. 58%) and week 100 (76% vs. 60%); all *p* < 0.05 (Fig. 4a). While substantial proportions of guselkumab-treated patients achieved both ER and SF-36 MCS response (range, 43%-48%), no association with ER status was observed at any time point (Fig. 4b).

Among guselkumab-treated patients achieving DR, sizable proportions achieved SF-36 PCS (range, 59–70%) and MCS (range, 46–52%) response over time, though no associations were observed between these outcomes and DR status (Fig. 4c and d).

## Discussion

Our findings indicate that guselkumab-treated patients achieving sustained ER and DR were more likely to experience meaningful improvements in key PROs through the end of the 2-year DISCOVER-2 study. Specifically, strong associations between ER and pain, physical function, and HRQoL responses were maintained while, to a lesser extent, DR was associated with improvements in fatigue, pain, and physical function over time. Furthermore, patients who achieved ER were significantly more likely to achieve DR and those achieving DR were more likely to achieve ER.

The observed association between ER and minimal pain response was consistent with previous findings establishing the direct relationship between enthesitis and pain [8, 9]. This relationship is noteworthy as pain can be difficult to treat and may prevent patients from achieving MDA (the accepted treatment target for PsA), thereby affecting overall well-being. Enthesitis present in lower regions of the body, such as the Achilles insertion and femur epicondyle, is more likely to be associated with higher levels of pain and fatigue and greater impairments in work and daily activity and HRQoL [9]. Although these are weight-bearing sites encountering higher levels of biomechanical stress that cannot be addressed by pharmacologic treatment, it is reassuring that associated pain was diminished in patients achieving enthesitis resolution with guselkumab [16, 34].

Both ER and DR were significantly associated with improvements in physical function, though those associations with both HAQ-DI response and normalized HAQ-DI were more apparent for ER. Similarly, in the current analyses and in previous reports utilizing pooled data from the DISCOVER-1 and -2 trials, a strong association between ER and the physical components of HRQoL was sustained through the end of the studies [23]. Such successful treatment of enthesitis with guselkumab would mitigate impairment in daily activities, thereby improving both function and HRQoL.

Fatigue is commonly reported by PsA patients and is a recognized contributor to disease burden, thereby emphasizing the need to improve fatigue as an important treatment goal [35, 36]. In patients with active PsA from the DISCOVER-1 and DISCOVER-2 studies, guselkumab 100 mg Q4W or Q8W led to clinically meaningful and sustained improvements in FACIT-Fatigue scores through 1 year [33]. Specifically, a greater proportion of guselkumab-randomized patients achieved such improvements compared with placebo at week 24 and demonstrated a clinically meaningful improvement in fatigue at week 52 across studies. We found that DR was associated with an increased likelihood of achieving fatigue response among biologic-naïve patients enrolled in DISCOVER-2, which is consistent with the findings from analyses previously conducted in the larger population of both biologic-naïve and -experienced patients with dactylitis in the pooled DISCOVER-1 and -2 population [22]. In that report, mediation analyses demonstrated the substantial direct effect of guselkumab on fatigue beyond its impact on achievement of MDA or 20% improvement in ACR response criteria or on changes in CRP levels. Taken together, results of both the previous and current analyses indicate that guselkumab’s impact on fatigue may be related to resolution of dactylitis. Further research is needed to identify the specific mechanism (e.g., improved mechanics of movement, effect of IL-23 inhibition on centralized pain, or normalization of fatigue-specific cytokine pathways) underlying treatment-related improvements in fatigue.

Approximately 35% of patients in the DISCOVER-2 population had both enthesitis and dactylitis at study outset. Interestingly, resolution of enthesitis with guselkumab was significantly associated with that of dactylitis and vice versa. These conditions are described as distinct phenotypes of PsA, with dactylitis (sausage digits) being related to flexor tenosynovitis and enthesitis being caused by inflammation at the attachment of tendons, ligaments, or joint capsules to bones [37]. However, one microanatomical study found that dactylitis may be a form of enthesitis involving small finger pulleys and fibrous sheaths [38]. Furthermore, another study found enthesitis and dactylitis were positively associated with the same MHC Class I genetic marker (i.e., the HLA-B*27:05:02) [39]. These commonalities may underly our finding that patients with DR were more likely to achieve ER (73%) than those without DR (52%) and vice versa (i.e., 88% and 75% with and without ER, respectively, achieved DR) at week 100.

Our findings confirm the efficacy of guselkumab for the treatment of enthesitis and dactylitis in patients with no prior biologic treatment. Specifically, decreases in LEI and DSS scores and rates of ER and DR were greater in guselkumab-treated patients compared with placebo at week 24, with response rates increasing among guselkumab-treated patients through 1 year and being maintained through 2 years [21]. Through the last efficacy assessment (week 100), two-thirds of guselkumab-treated patients achieved ER and three-quarters achieved DR. These results are similar to those based on pooled analyses through 1 year in subpopulations with enthesitis and/or dactylitis in both DISCOVER studies as well as the phase 2 study of guselkumab in PsA, which included patients with and without prior biologic experience [22, 23, 40]. The robust efficacy of guselkumab reported here further highlights the central role of the IL-23/IL-17 immune axis in the pathogenesis of PsA. It also validates the p19 subunit of IL-23 as a relevant target for therapeutic intervention in patients with enthesitis and dactylitis. Results from other studies of patients treated with guselkumab and other biologic agents targeting pro-inflammatory cytokines in the IL-12, IL-23 or IL-17 pathways also confirm the appropriate use of guselkumab to treat these specific disease domains [22, 23, 41].

These analyses were limited; they were largely post hoc in nature and patient cohorts with and without specific disease domains (especially enthesitis) were imbalanced in size. The presence of enthesitis and dactylitis was assessed using the LEI and DSS, rather than imaging techniques capable of capturing inflammatory changes [2, 3], and the high prevalence of overweight and obese patients (72%) enrolled into these trials may have influenced the assessment of these features [7, 42]. The LEI evaluates a limited number of anatomical sites (*n* = 6) with enthesitis, though results using this measure are concordant with other indices. Objective determination of the resolution of enthesitis is complicated by factors including chronic pain, fibromyalgia, and mechanically associated enthesopathy; thus, it is possible that the degree of true inflammatory enthesitis resolution was even greater.

Only biologic-naïve patients were included, which may limit the generalizability of the results for patients treated previously with other biologic agents; however, studies of pooled DISCOVER-1 and DISCOVER-2 data support some of the same associations [22, 23]. While analyses were not performed to evaluate the consistency of the relationships across subgroups defined by demographic or disease characteristics, in previous analyses from DISCOVER-2, rates of achieving resolution of dactylitis and enthesitis were largely consistent across such subgroups through 2 years [43]. Owing to the limited sample size of patients with both enthesitis and dactylitis at baseline, the analyses examining the associations between concurrent ER/DR and PRO improvements were not performed in this smaller patient subgroup. However, given that the overall demographic and baseline disease characteristics, including PRO assessments, were generally similar between patients with both enthesitis or dactylitis and those with either condition, it is expected that patterns would be consistent. Finally, other factors beyond ER or DR that may have affected PROs were not considered. For example, the presence of chronic pain related to factors other than enthesitis (e.g., fibromyalgia, residual joint inflammation) and the direct effect of improvements in dactylitis and enthesitis apart from improvements in other PsA domains cannot be differentiated in this analysis. As a strength, the similar NSAID use in patients with and without enthesitis at baseline indicates findings were not confounded by differential effects on prostaglandin-2-mediated pathways believed to play a pathophysiological role [6].

## Conclusion

These results indicate that ER and DR with guselkumab treatment may also lower the overall burden of disease as measured by meaningful responses in fatigue, pain, function, and HRQoL in PsA patients. While improvement in PROs would be expected to accompany resolution of these important aspects of disease, the distinct effects of the two manifestations on specific patient outcomes are noteworthy. Future studies will provide insight into potential mechanisms impacting PROs and determining treatment choices by PsA phenotype.

### Supplementary Information

Below is the link to the electronic supplementary material.

Supplementary file1 (DOCX 1697 KB)

## Data Availability

The data sharing policy of Janssen Pharmaceutical Companies of Johnson & Johnson is available at https://www.janssen.com/clinical-trials/transparency. As noted on this site, requests for access to the study data can be submitted through Yale Open Data Access (YODA) Project site at http://yoda.yale.edu.
