# Supplementary material for: Association between enthesitis/dactylitis resolution and patient-reported outcomes in guselkumab-treated patients with psoriatic arthritis
Source: Clin Rheumatol. 2024 Mar 12;43(5):1591–604. doi: 10.1007/s10067-024-06921-8 (PMC11018666; doi:10.1007/s10067-024-06921-8)

**SUPPLEMENTAL DATA**

**Supplemental Fig. 1** LS mean change (95% CI) from baseline over time in **a**) LEI and **b**) DSS in patients with enthesitis or dactylitis by treatment group at weeks 24, 52, and 100. CI, confidence interval, DSS, Dactylitis Severity Score; GUS, guselkumab; LEI, Leeds Enthesitis Index; LS, least squares; PBO, placebo; Q4W, every 4 weeks; Q8W, every 8 weeks


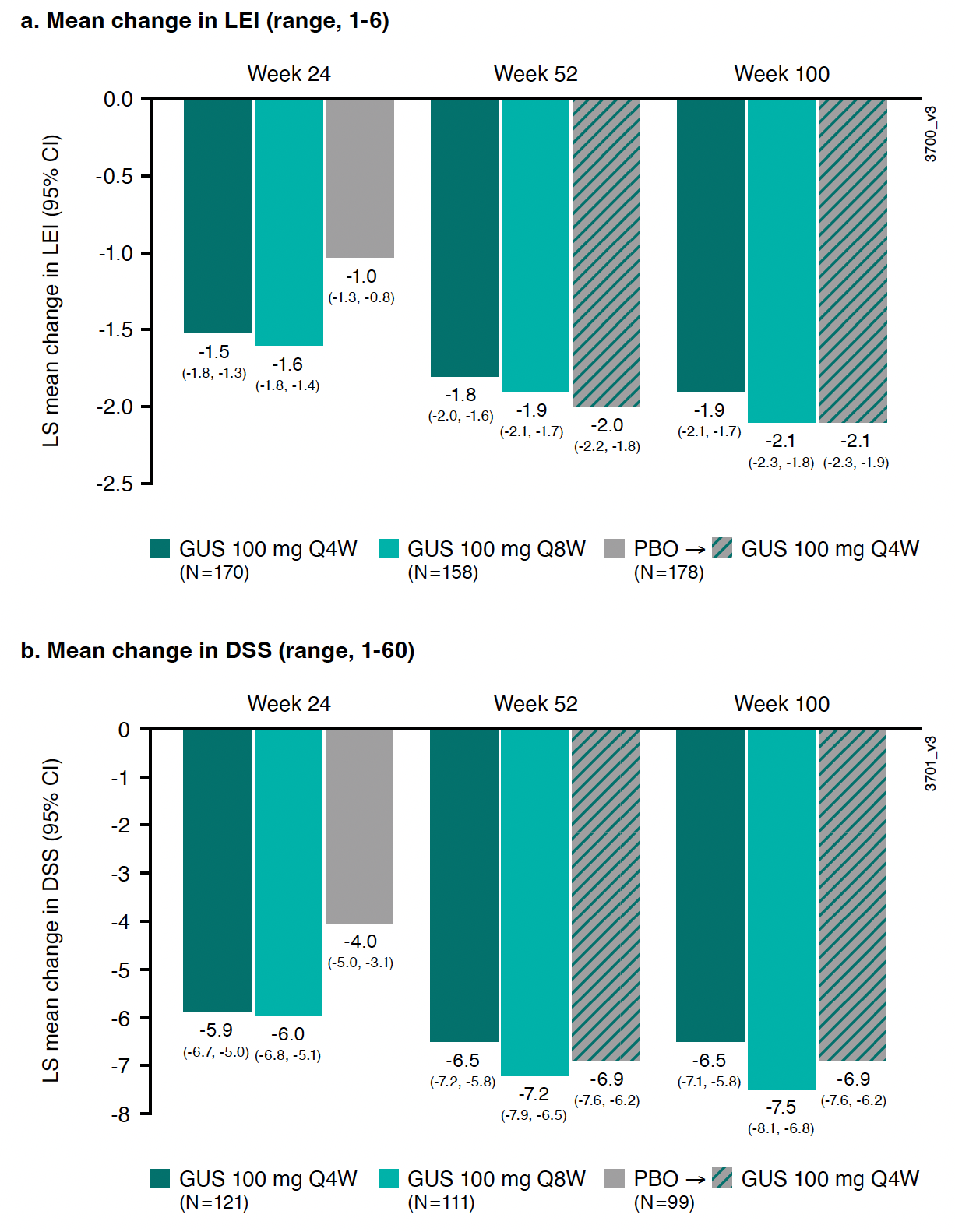


**Supplemental Fig. 2** Proportion of patients achieving **a**) ER and **b**) DR over time. DR, dactylitis resolution; DSS, Dactylitis Severity Score; ER, enthesitis resolution; GUS, guselkumab; LEI, Leeds Enthesitis Index; PBO, placebo; Q4W, every 4 weeks; Q8W, every 8 weeks


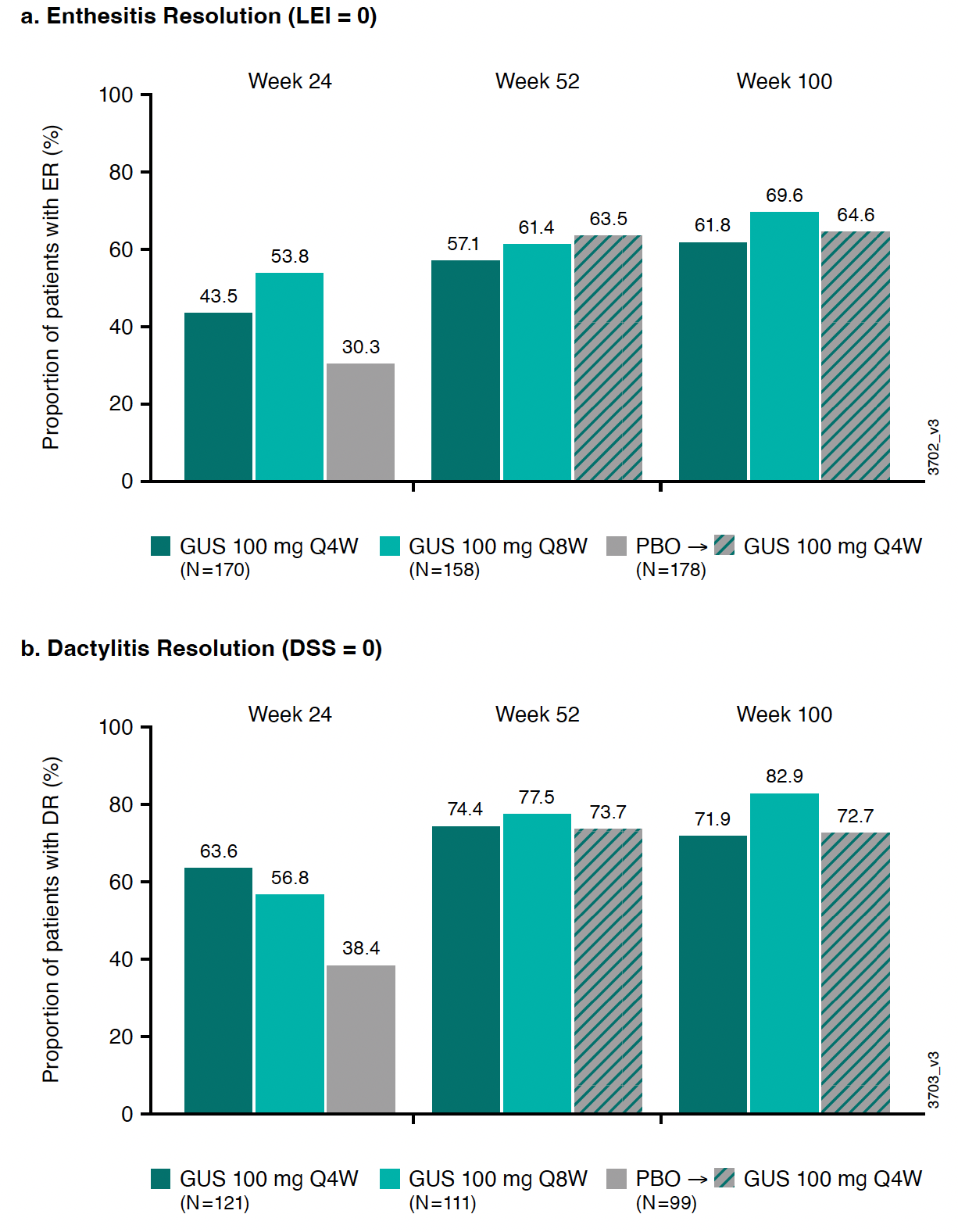

Supplement: Supplementary file 1 — Supplementary file1 (DOCX 1697 KB) [file 10067_2024_6921_MOESM1_ESM.docx]
